# Medical Items

**Published:** 1918-01

**Authors:** 


### MEDICAL ITEMS.

### SILVOL: A USEFUL GERMICIDE.

The signal property of Silvol is its germicidal and non-irritating effect on inflamed and infected mucous membranes. This notable proteid-silver contains approximately 20 per cent. of metallic silver. It is non-toxic. It is freely soluble in water, and its solutions do not require filtration. It does not coagulate albumin, and it is not precipitated by proteids or alkalies. It stains less than other proteid-silver combinations.

Silvol may be used in the treatment of infected and inflamed mucous membranes in strengths ranging from 2 to 50 per cent. It has been suggested that the addition of a small proportion of gylcerin to Silvol solutions will prevent them from spreading too freely over the surfaces to which they are applied; that the glycerinized solution forms a thin film over the surface that does not immediately dry off. In other words the glycerin ''holds'' the Silvol to the spot.

Silvol has been successfully used in the treatment of nasal and pharyngeal inflammations, both acute and chronic. One practitioner reports his experience with a 20 per cent. solution of Silvol in over five hundred cases of various nasal, laryngeal and ear diseases. The solution was used either as a spray, instillation, or topical application.

An eastern specialist reports gratifying success with Silvol in the treatment of twenty-five cases of antral infection. The treatment consisted in first irrigating the sinus with normal salt solution, then injecting it as nearly full as possible with a 10 per cent. solution of Silvol, repeating daily. Usually the cases cleared up in less than a week, often within three or four days. Another writer observed most striking results in tracheitis and bronchial asthma. In these cases a 10 per cent. solution was applied twice a week.

A prominent urologist reports the successful use of Silvol in one hundred cases of posterionr urethritis, gonorrheal in origin. A 10 per cent. solution was applied, once a day, to the posterior urethra with an Ultzmann syringe, and each patient received a supply of the same solution for home use, to be applied two or three times a day.

Results from the use of Silvol in the treatment of eye diseases are highly favorable. A well-known opthalmologist says: "My clinical observations of Silvol evidenced to me that in all forms of acute conjunctivitis, acute epidemic to acute gonorrheal, this product is a powerful bactericide, apparently absolutely non-irritating; even in a solution of 50 per cent. used as frequently as every fifteen minutes, day and night, for a period of three days."

An experienced surgeon makes the assertion that Silvol is a better application in ophthalmia of the new-born than silver-nitrate. It can be used with less risk and the writer thinks he can control any case with it.

Silvol is supplied in the following forms: Ounce bottles; six-grain capsules, bottles of fifty; Vaginal Suppositories of Silvol, 5 per cent., boxes of one dozen; Silvol Ointment, 5 per cent., collapsible long-muzzled tubes, two sizes; Silvol Bougies, 5 per cent., boxes of 25 and 100.

---

### THE MOST EFFECTIVE THERAPEUTIC REMEDY FOR NERVOUS DISEASES.

In the treatment of many nervous diseases it not infrequently happens that the after-effects, or sequelae of the remedy are worse than the disease. his statement applies in particular to the bromides in the forms commonly employed. Valuable and effective as they are whenever a sedative or antispasmodic is needed, they must be prescribed with the utmost care and discretion, else the results, to put it mildly, may be unfortunate in the extreme.

The physician therefore, cannot be too critical and cautious in selecting the preparation of the bromides he uses, especially if it must be given for any considerable period. Many men know the advantages of Peacock's Bromides but to those who do not is should be pointed out that this high grade product easily stands at the head of available bromide prepaartions not only in freedom from objectionable effects but also in therapeutic efficiency.

Extended clinical experience has shown that owing to the purity and quality of the constituent salts, this combination does not upset the organs of digestion, nor give rise to the highly disagreeable condition known as bromism.

Physicians who use Peacock's Bromides know the satisfaction of accomplishing not only the results they seek, but without creating any disagreeable and annoying conditions which call for explanation and excuse.

### AFTER AN ALCOHOLIC DEBAUCH.

The physician is often caled upon for relief of the marked nervous disturbance following a prolonged indulgence in al- stomashrdlu estaoin shrdlu cmfwyp SHRDLU LUUUUUUY coholic liquors. The nervous system is badly upset, the stomach is disturbed and the patient is unable to perform his usual duties. In the relief of this state PASADYNE (Daniel( shows its advantages in a high degree. Possessing marked sedative properties it quckly and safely secures for the pation the rest needed and restores the nervous system to his wonted equilibrium. It may be pushed without fear of produsing a depressing reaction, and its therapeutic potency insures accomplishment of the prime purpose of its administration.

PASADYNE (Daniel) is a concentrated tincture of passiflora incarnata, and is a calmative of the highest order. It is of particular value in women.

A sample bottle may be obtained by addressing the laboratory of John B. aDniel, Inc., Atlanta, Ga.

---

### BUILDING UP AFTER PNEUMONIA.

Every physician recognizes that one of the most important things in the management of a case of pneumonia is building u pthe patient after the resolution of the inflammatory process. For this purpose Cord. Ext. Ol. Morrhuae Comp. (Hagee) has been found of great dependable worth, owing to the certainty with which it charges the reduced tissues with the necessary reconstructive elements. It is a most rational therapeutic procedure to put the pneumonia patient on Cord. Ext. Ol. Morrhuae Comp. (Hagee) for by so doing the period of convalescence is shortened and the patient is restored to his normal vigor all the more quickly. The advantage of using Cord. Ext. Ol. Morrhuae Comp. (Hagee) is that while it possesses every remedial and nutritive quality of the plain oil, it inflicts no burden on the gastric apparatus, hence it may be given for an indefinite time without causing distress.

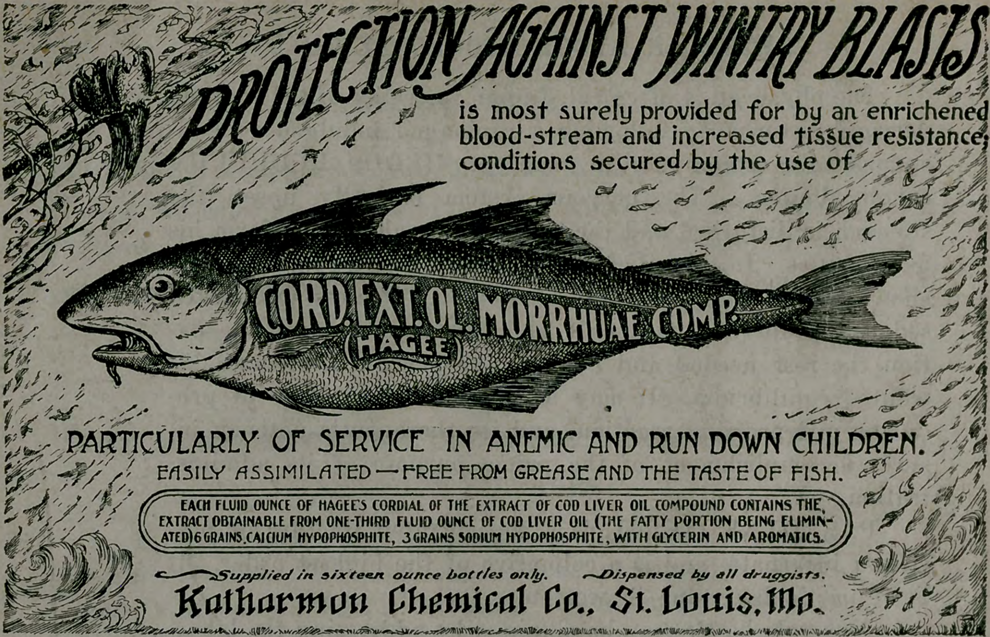

***KATHARMON** reduces pneumonia susceptibility if used as a mouth and throat wash. Of distinctive value in mucous membrane inflammations.*

KATHARMON CHEMICAL CO., ST. LOUIS, MO.

KATHARMON represents in combination Hydrastis Canadensis, Thymus Vulgaris, Mentha Arvensis, Phytolacca Decandra, 10½ grains Acid Borosalicylic, 24 grains Sodium Pyroborate to each fluid ounce of Pure Distilled Extract of Witch Hazel.

---

### THE TREATMENT OF EPILEPSY.

In the treatment of epilepsy the employment of the bromides is of such universal acceptance that the main point involved is the choice of a bromide preparation that will subject the patient to a minimum of the evil effect of the bromides.

Many physician have shown that the best preparation to use for this purpose, one that has a maximum of therapeutic potency and productive of a minimum of the gastric disturbances so frequent in the long continued use of bromides, is BROMIDIA (Battle). This superiority of BROMIDIA (Battle) is due to the choosing of pure drugs and the employment of the utmost care adn skill in compounding it.

BROMIDIA (Battle) may be continued for long periods, a feature that gives it preeminence in the treatment of epilepsy.

